# Zero fluoroscopy ablation for atrioventricular nodal reentrant tachycardia and typical atrial flutter is equally safe and effective with EnSite NavX, Carto3, and Rhythmia mapping systems

**DOI:** 10.3389/fcvm.2023.1185187

**Published:** 2023-07-25

**Authors:** Katalin Piros, Péter Perge, Zoltán Salló, Szilvia Herczeg, Vivien Klaudia Nagy, István Osztheimer, Béla Merkely, László Gellér, Nándor Szegedi

**Affiliations:** Cardiology Department, Heart and Vascular Center, Semmelweis University, Budapest, Hungary

**Keywords:** zero fluoroscopy, catheter ablation, EnSite NavX, CARTO3, Rhythmia

## Abstract

**Purpose:**

Our purpose was to compare the procedural characteristics, success rate, and complication rate of the conventional fluoroscopic (CF) and the zero-fluoroscopic (ZF) approach in patients undergoing catheter ablation of AVNRT or typical atrial flutter (Aflu).

**Methods:**

186 consecutive patients with an indication for AVNRT or Aflu ablation were enrolled. Based on the operator's preference, the patients were assigned to either CF or ZF group. In the ZF group EnSite NavX, Carto3, or Rhythmia EAMS were used for catheter guidance.

**Results:**

The median age was 56 (IQR = 42−68) years, 144 patients had AVNRT, and 42 had Aflu ablation. CF approach was chosen in 123 cases, while ZF in 63 cases. ZF approach was used more often in case of AVNRT patients [56 (39%) vs. 7 (17%), *p* = 0.006] and in the case of female patients [43 (68%) vs. 20 (32%), *p* = 0.008]. Acute procedural success was obtained in all cases. There was no difference in the complication rate (1 vs. 1, *p* > 0.99) between the two groups. No difference was found regarding the procedure time between the CF and ZF groups [CF: 55 (46–60) min, ZF 60 (47–65) min; *p* = 0.487] or in the procedure time for the different EAMS [EnSite NavX: 58 (50–63) min, Carto3: 60 (44.5–66.3) min, Rhythmia: 55 (35–69) min; *p* = 0.887]. A similar success rate was seen at the 3-month follow-up in the two groups [41 (100%) vs. 96 (97%); *p* = 0.55].

**Discussion:**

The ZF approach demonstrated non-inferiority in safety and efficacy compared with CF for the AVNRT and Aflu ablations.

## 1. Introduction

Catheter ablation is recommended as the first-line therapy for recurrent, symptomatic, or persistent typical atrial flutter (Aflu) and atrioventricular nodal reentrant tachycardia (AVNRT) according to the current guideline (1). Traditionally, conventional electrophysiological procedures require the use of radiation to visualize catheter positions. Due to the stochastic effect of radiation, even a low dose of fluoroscopy can be harmful; thus, no safe dose exists (2, 3). The ALARA (“As Low As Reasonably Achievable”) concept was created as an essential step toward minimizing ionizing radiation used in the diagnostic and therapeutic approaches (4). Besides the x-ray shields and lead aprons, other protecting devices can be used to care for the medical staff. However, these heavy protectors may lead to a variety of musculoskeletal disorders (5). Though, in an Italian study, it was shown that the risks of ionizing radiation are well-known among practitioners, and it has been raised in recent years, awareness of risk is key in applying the ALARA principle (6). Carpeggiani et al. prove that radiation risk awareness can be easily improved with limited teaching effort through targeted training (7).

The fluoroscopy usage differs in a wide range among different EP labs (8). With the development of electroanatomical mapping systems (EAMS), diagnosis, mapping, and ablation of tachyarrhythmias can be performed with lower ionizing radiation doses. The NO-PARTY multicenter randomized trial confirmed that the use of the EnSite NavX (St. Jude Medical, Inc., St. Paul, MN, USA) mapping system during supraventricular tachycardia (SVT) ablation leads to a significant reduction of radiation exposure, which might result in a dramatic risk reduction of cancer and mortality (9). Also, we know that zero fluoroscopic approaches can be used with both radiofrequency and cryo energy (10).

The zero-fluoroscopic approach arouses high scientific and clinical interest. Diverse data is available regarding near-zero fluoroscopy SVT ablation procedures using Carto3 (Biosense Webster, Inc., Baldwin Park, CA, USA) and EnSite NavX EAMS (5, 8, 9, 11). However, limited data exist regarding the complete zero-fluoroscopy approach, especially with the Rhythmia electroanatomical mapping system (Boston Scientific, Natick, MA, USA).

We aimed to compare the procedural characteristics, acute success rate and success rate at 3-month follow- up, and complication rate of the conventional fluoroscopic (CF) and the zero-fluoroscopic (ZF) approach in patients undergoing catheter ablation for AVNRT or Aflu in our high volume center.

## 2. Methods

### 2.1. Study population

We enrolled 186 consecutive patients who underwent radiofrequency catheter ablation for AVNRT or Aflu at the Heart and Vascular Center of Semmelweis University, Budapest, Hungary, between July 2018 and July 2020 in the study. Inclusion criteria were documented, symptomatic Aflu or AVNRT. Patients without ECG documentation and patients who were previously enrolled in other clinical trials were excluded. Participants were assigned to either the ZF or CF group according to the operator's preference. EAMS were not used in the CF group. However, the use of fluoroscopy was allowed in the ZF group whenever it was necessary. Procedures were performed by 4 experienced operators, who were familiar with using all three mapping systems. Anthropometric characteristics, relevant medical history, and current medical therapy of the patients were collected. Structural heart disease was defined as the presence of ischemic or valvular heart disease or heart failure. The efficacy endpoint was acute success, defined as non-inducibility in case of the AVNRT (which was inducible before the ablation) and bidirectional cavo-tricuspid isthmus (CTI) block in case of the Aflu. The safety endpoint was the occurrence of periprocedural complications. Fluoroscopy dose was calculated using the internationally used formula: ED (mSv) = KAP (Gycm2) × 0.2 (mSv/Gycm2) (11). All patients provided written informed consent to the ablation procedure, data retrieval, and analysis. The study protocol was reviewed and approved by the Regional and Institutional Committee of Science and Research Ethics of Semmelweis University (No: 179/2020), and was in accordance with the Declarations of Helsinki.

### 2.2. Catheter ablation procedure

Femoral venous access was used for all procedures. In AVNRT patients, quadripolar catheters were placed in the high right atrium (HRA), the right ventricular apex (RVA), and a decapolar catheter was inserted into the coronary sinus. After the electrophysiological diagnostic maneuvers, the HRA catheter was replaced by a non-irrigated 4 mm tip ablation catheter. Radiofrequency ablations in the slow pathway region were applied in temperature control mode (65°C temperature limit, 40 W power).

In Aflu patients, a decapolar catheter was inserted into the coronary sinus, and a Halo catheter was placed in the right atrium (around the tricuspid annulus). An irrigated 4 mm tip ablation catheter was introduced to the cavo-tricuspid isthmus region, where radiofrequency energy was applied in temperature control mode (43°C temperature limit, 40 W power).

In the CF group, diagnostic catheters were introduced under x-ray guidance into the heart and positioned in the above-described positions. Ablation was performed under fluoroscopy guidance as well. Obviously, to limit the dose of ionizing radiation, fluoroscopy was only used during the procedure when it was essential, according to the ALARA principle.

**Figure 1:**
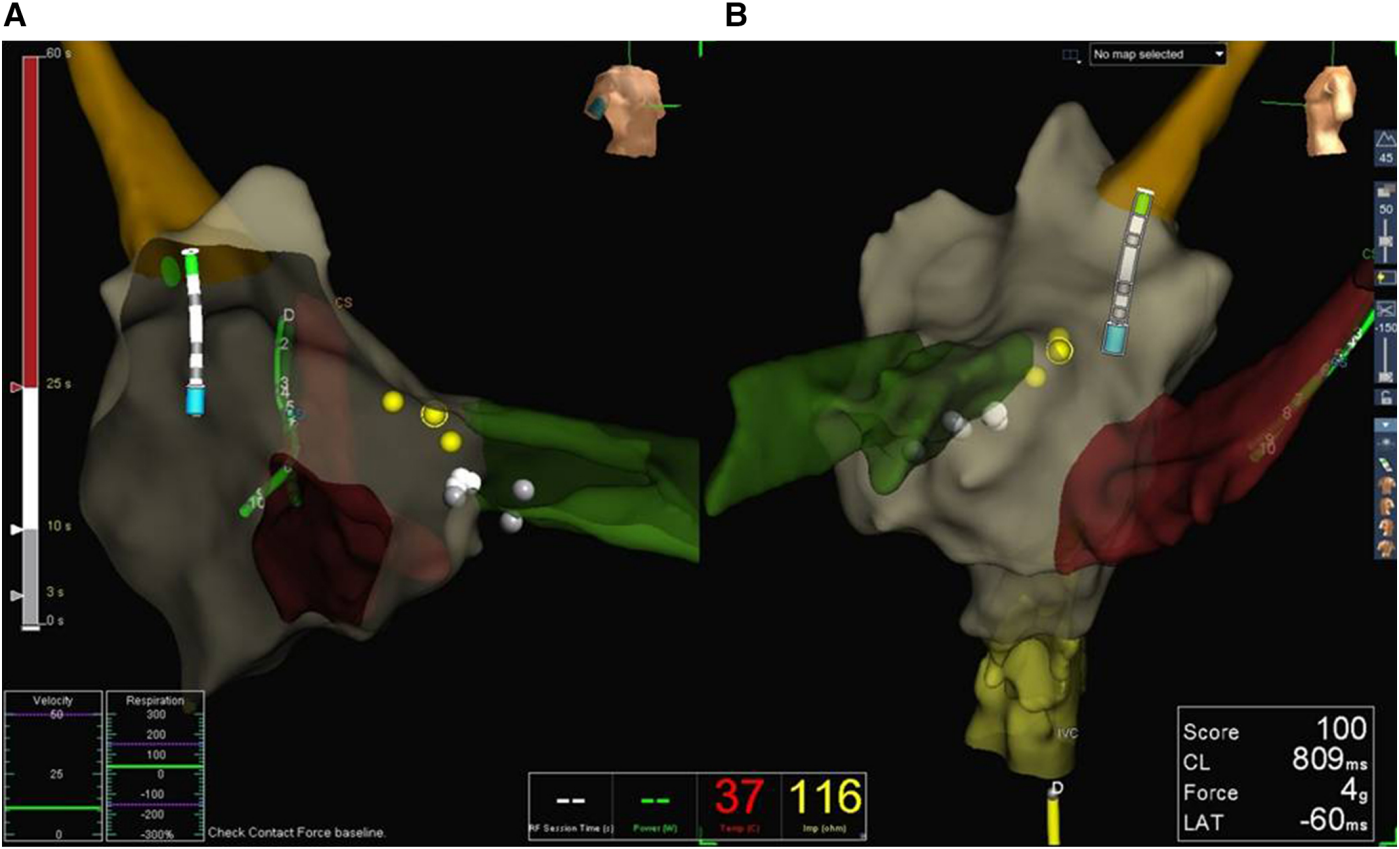
Electroanatomical map created with the EnSite NavX system in a patient with AVNRT. Right anterior oblique (RAO, **A**) and left anterior oblique (LAO, **B**) views are shown. A decapolar mapping catheter is inserted in the coronary sinus, and an ablation catheter is placed in the superior vena cava. The yellow dots represent the His region, while the white dots are the ablation points.

During the ZF procedures performed with the EnSite NavX system, a decapolar diagnostic catheter (Dynamic XT Steerable Diagnostic Catheter) was introduced as the first step, and an anatomical map of the venous route was created. After respiration compensation, mapping of the right atrium, the right ventricle, and the coronary sinus were performed. Two diagnostic quadripolar catheters (MultiCath 4 pole diagnostic catheter) were introduced and positioned in their specific positions; meanwhile, the map was further created. Catheter ablation was guided by the EnSite NavX EAMS without fluoroscopy. In AVNRT patients, His region was tagged before slow pathway ablation (Figure 1).

**Figure 2:**
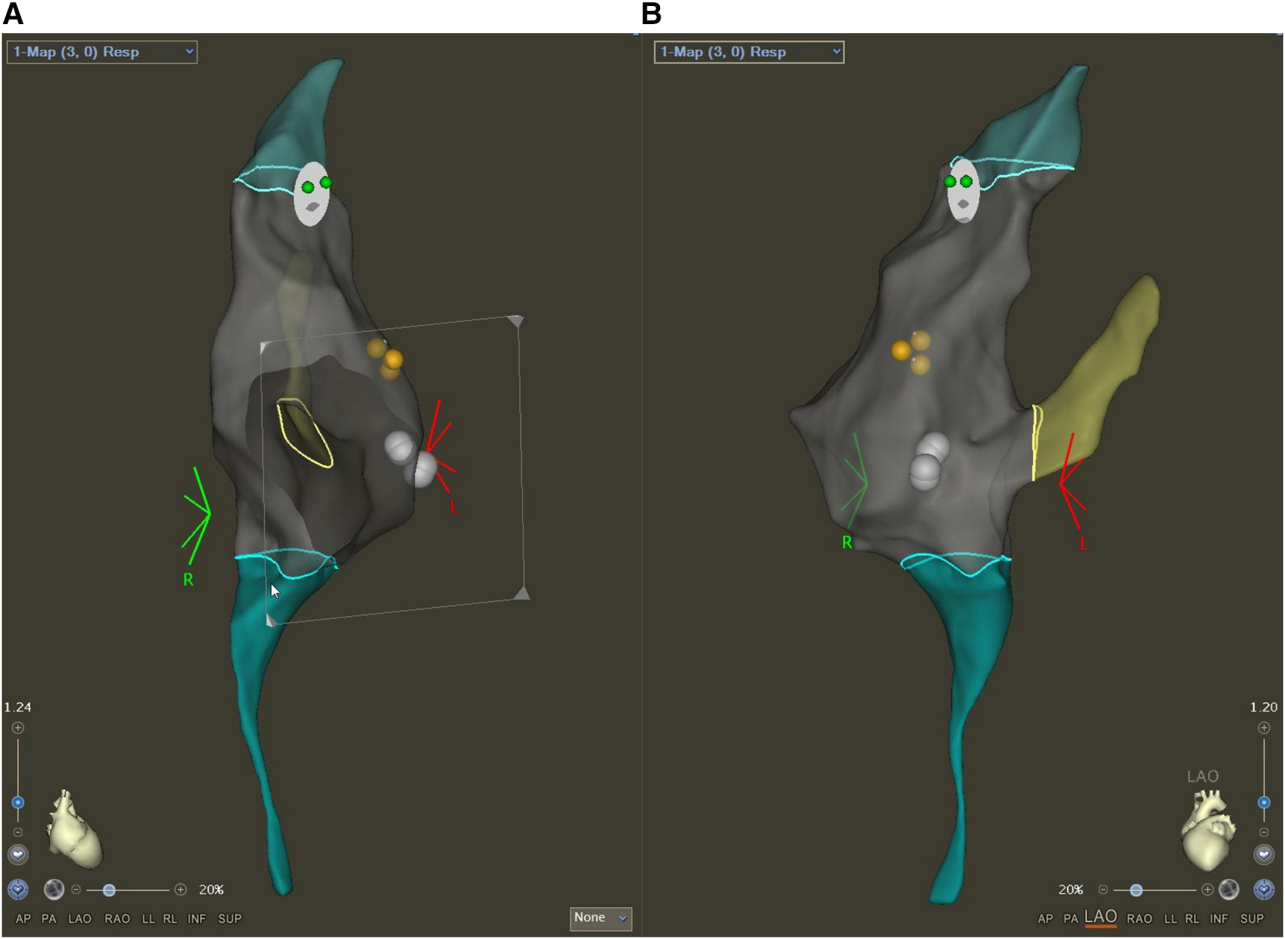
Electroanatomical map created with the Carto3 system in a patient with AVNRT. Right anterior oblique (RAO, **A**) and left anterior oblique (LAO, **B**) views are shown. The yellow tags indicate the His region, while the white dots represent the ablation points.

**Figure 3:**
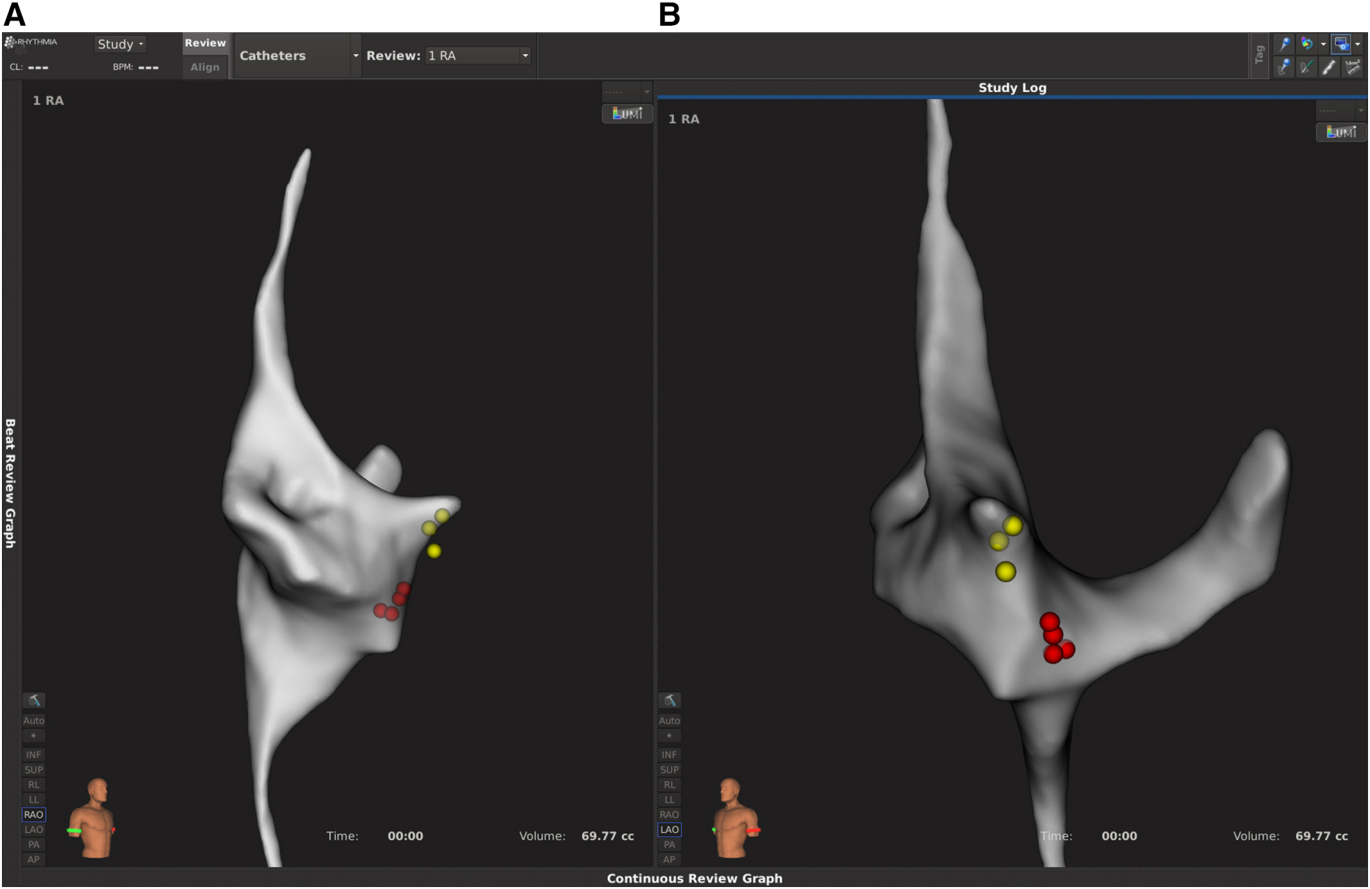
Electroanatomical map created with the Rhythmia system in a patient with AVNRT. Right anterior oblique (RAO, **A**) and left anterior oblique (LAO, **B**) views are shown. The yellow dots represent His region, while the red dots indicate the ablation points.

In the case of procedures performed with Carto3 (Figure 2) or Rhythmia (Figure 3) systems, the ablation catheter (NaviStar catheter; Intella NAV XP Ablation Catheter) was introduced first, and an electroanatomical map of the right atrium, the right ventricle, and the coronary sinus were created. Further diagnostic catheters (Dynamic XT Steerable Diagnostic Catheter; Halo XP Tricuspid Mapping Catheter; MultiCath 4 pole diagnostic catheter) were positioned using the previously created map. The following steps of the procedure were similar to the EnSite NavX procedures.

### 2.3. Statistical analysis

The majority of the variables showed non-parametric distributions after performing the Shapiro-Wilk test. The continuous variables were expressed as medians and interquartile ranges. Categorical variables were expressed as percentages with event numbers. Continuous variables were compared with the Mann-Whitney test and the Kruskal-Wallis test, respectively. Fisher's exact test was applied for categorical data comparisons.

A two-tailed *p*-value of <0.05 was considered statistically significant. Statistical analyses were performed using IBM SPSS 25 (Apache Software Foundation, USA) and GraphPad Prism 8.01 (GraphPad Software, Inc., USA) software products.

## 3. Results

We included 186 patients in our analysis. The median age was 56 (42-68) years; 46% of patients were male. CF and ZF groups consisted of 123 and 63 patients, respectively. AVNRT ablation was performed in 144, while Aflu ablation in 42 patients. Antiarrhythmic drugs were used prior to the procedure in 23% and 13% of patients in the CF and ZF group, respectively (*p* = 0.167). No difference was found in the baseline characteristics between the CF and the ZF group (Table 1). The ZF approach was used more often in case of AVNRT compared to the Aflu [56 (39%) vs. 7 (17%), *p* = 0.0058]. Also, the ZF approach was utilized more commonly in case of female patients compared to males [43 (68%) vs. 20 (32%), *p* = 0.008]. Among ZF procedures, the EnSite NavX, Carto3, and Rhythmia systems were used in 41 (65%), 14 (22%), and 8 (13%) cases, respectively.

**Table 1:** Baseline characteristics of the study population.

	CF group (*n* = 123)	ZF group (*n* = 63)	*P*-value
Female	58 (47%)	43 (68%)	0.008*
Age	57 [42–69]	56 [38–65]	0.371
SHD	13 (11%)	2 (4%)	0.151
Hypertension	57 (46%)	23 (37%)	0.274
Diabetes mellitus	29 (23%)	16 (25%)	0.856
Pre-procedural AAD therapy	28 (23%)	8 (13%)	0.167

AAD: antiarrhythmic drug; CF: conventional fluoroscopy; SHD: structural heart disease; ZF: zero-fluoroscopy.

Data is expressed as median with interquartile range for continuous variables and as event numbers with percentage for categorical variables. Data are compared with the Fisher's exact test and the Mann-Whitney test.

When examining the zero-fluoro groups separately, we found that in the Ensite group, the patients tend to be older, and more patients had hypertension (*p* = 0.007, *p* = 0.024).

*clinically significant.

Acute procedural success was achieved in 100% of the patients in both groups (*p* > 0.99). We had one complication in each group (*p* > 0.99). In the CF group, one transient right bundle branch block occurred. In the ZF group, one inguinal hematoma was observed, which did not require any invasive treatment. Both patients resolved without sequelae. There was no difference in the success rate at 3-month follow-up between ZF and CF groups [41 (100%) vs. 96 (97%); *p* = 0.55].

**Figure 4:**
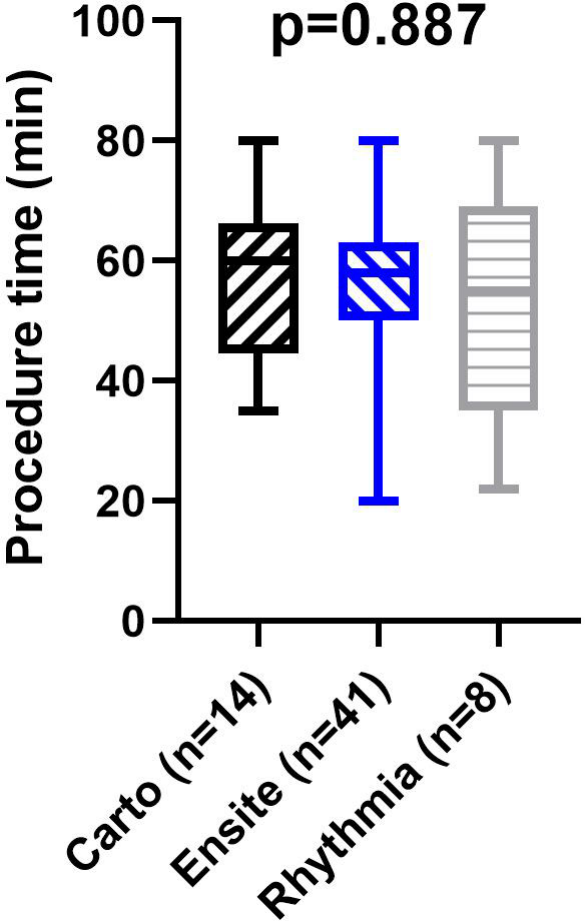
Procedure times of the zero fluoroscopy ablations performed with the different mapping systems. Procedure times were compared with the Kruskal-Wallis test, ns: *p* > 0.05.

Fluoroscopic time, fluoroscopic dose, and effective dose were significantly higher in the CF group. No difference was found in the procedure time between the two groups (Table 2). Within the ZF group, there was no difference in the procedure time regarding the different EAMS [EnSite NavX: 58 (50-63) min, Carto3: 60 (44.5-66.3) min, Rhythmia: 55 (35-69) min; *p* = 0.887] (Figure 4). Totally zero fluoroscopy was achieved in 62 (98.4%) out of 63 patients in the ZF group. We had a conversion to fluoroscopy in one case, when Ensite EAM was used initially to perform the diagnostic electrophysiology tests. However, due to difficulties during the ablation catheter's introduction we decided to use fluoroscopy to enhance the catheter guidance. During this procedure, a total fluoroscopy dose was 0.058 mGym2; the fluoroscopy duration was 39 s.

**Table 2:** Fluoroscopy and procedural parameters of the study population.

	CF group	ZF group	*P*-value
Fluoroscopy time (sec)	104 [69–192]	0 [0–0]	<0.0001*
Fluoroscopy dose (mGym2)	0.12 [0.071–0.219]	0 [0–0]	<0.0001*
Effective dose (mSv)	0.22 [0.141–0.435]	0.12^a^	<0.0001*
Procedure time (min)	55 [46–60]	60 [47–65]	0.487

CF: conventional fluoroscopy; ZF: zero-fluoroscopy.

Characteristics of the groups were compared with the Mann-Whitney test.

* clinically significant.

^a^ There was only one case where fluoroscopy was used in the ZF group.

## 4. Discussion

### 4.1. Key findings

According to our results, the ZF ablation approach is equally safe and effective as the CF ablation approach for the treatment of AVNRT and typical atrial flutter. Our study is the first to investigate all the three widely used EAMS for total zero-fluoroscopy SVT ablations. Furthermore, we observed no difference in procedure times when comparing EnSite NavX, Carto3, and Rhythmia systems.

### 4.2. Zero fluoroscopy ablation for supraventricular tachycardias with EAMS

Positive effects of the avoidance of fluoroscopy are well-known both for the patient and for the operating team (2, 4, 5). Our results correspond with previous experiences, where the ZF approach proved to have similar safety and efficacy as the CF approach (12–20).

Casella et al. and Giaccardi et al. demonstrated that the ablation of supraventricular tachycardias can be performed safely and effectively using the EnSite NavX system without fluoroscopy (12, 20). A multicenter, non-randomized study also demonstrated that using the EnSite NavX system during zero-fluoroscopy ablations is equivalently safe and effective as the conventional approach (14). Macias et al. showed that the Carto3 electroanatomical mapping system provides the same safety, feasibility, and efficacy even at a 3-month follow- up as using the EnSite NavX system for the ablation of typical atrial flutter (17). Fadhle et al. found no significant difference in the safety and efficacy of fluoroscopic- and non-fluoroscopic approaches. They also did not find any difference in the outcomes comparing Carto3- and EnSite-based procedures. Similar to our study, they found 100% midterm success rate in ZF group (15). The long-term efficacy of SVT ablation without fluoroscopy was also proven in a single-center, observational study, which promotes the effort to perform electrophysiological interventions with EAMS (21). Based on the results of a preliminary study, there is no difference in the acute success rate or complication rate between the CF approach or ZF approach using the Rhythmia system. However, the study included only ten patients in the Rhythmia arm, and there were conversions to fluoroscopy in 2 out of 10 cases (13).

All the above mentioned studies were limited by comparing maximum of two mapping systems. Our research group was the first to include all three widely used EAMS in the analysis. Additionally, to the benefit of decreasing the estimated lifetime risk of malignancy for both the patient and the medical team (9), the risk of musculoskeletal diseases of the medical team should also be taken into consideration. Furthermore, zero fluoroscopy permits the ablation of unique populations, e.g., pregnant patients or patients receiving radiation therapy. Additionally, using EAMS contribute to a more precise location of the arrhythmia substrate, with the ability to depict activation sequence. It also allows a more precise catheter manipulation by marking certain points during the procedure (22).

In our cohort, the ZF approach was used more often in AVNRT patients, which might be explained by the higher proportion of females in the ZF group as AVNRT is more frequent in females (1). Although the use of EAMS increases the procedure costs, this seems to be comparable with the expenses resulting from the consequences of diverse radiation-induced diseases (9).

Based on our results, zero-fluoroscopy procedures with electroanatomical mapping system guidance could be advised for every Electrophysiology Laboratory, as it seems to be safe and effective in the diagnosis and treatment of arrhythmias with the obvious benefits of reducing or abolishing ionizing radiation.

## 5. Conclusions

All three EAMS are equally appropriate for catheter navigation during the zero-fluoroscopic ablation of AVNRT and typical atrial flutter. There was no difference in the acute success rate and complication rate of zero-fluoroscopic procedures compared to conventional procedures.

## Limitations

The main limitation of our study is the single-center observational layout. Another limitation is the relatively small sample size. Furthermore, the effective dose was estimated from the dose area product using correcting coefficients, which provides only a rough estimation of the effective dose.

## Data Availability

The original contributions presented in the study are included in the article/**Supplementary Material**, further inquiries can be directed to the corresponding author.
