# Supplementary material for: Zero fluoroscopy ablation for atrioventricular nodal reentrant tachycardia and typical atrial flutter is equally safe and effective with EnSite NavX, Carto3, and Rhythmia mapping systems
Source: Front Cardiovasc Med. 2023 Jul 25;10:1185187. doi: 10.3389/fcvm.2023.1185187 (PMC10407085; doi:10.3389/fcvm.2023.1185187)
Supplement: Supplementary file 1 [file Table1.docx]

The distrubution of patients using different approaches for ablation of SVT.
